# Jian-Pi-Yi-Shen Decoction Relieves Renal Anemia in 5/6 Nephrectomized Rats: Production of Erythropoietin via Hypoxia Inducible Factor Signaling

**DOI:** 10.1155/2019/2807926

**Published:** 2019-03-03

**Authors:** Jianping Chen, Fochang Wang, Shiying Huang, Xiaoyan Liu, Zhonggui Li, Airong Qi, Xinhui Liu, Tiegang Yi, Shunmin Li

**Affiliations:** ^1^Shenzhen Key Laboratory of Hospital Chinese Medicine Preparation, Shenzhen Traditional Chinese Medicine Hospital, The Fourth Clinical Medical College of Guangzhou University of Chinese Medicine, Shenzhen, China; ^2^Department of Nephrology, Shenzhen Traditional Chinese Medicine Hospital, The Fourth Clinical Medical College of Guangzhou University of Chinese Medicine, Shenzhen, China

## Abstract

Jian-Pi-Yi-Shen (JPYS) is one of the herbal medicines for treatment of anemic patients with chronic kidney disease (CKD). However, less of scientific evidence to support JPYS involved in treating anemia has been revealed. Here, an animal study was performed to investigate its hematopoietic activities and the underlying mechanism. The 5/6 nephrectomized inductions of CKD anemic rats were randomly divided into two groups: CKD group and JPYS group. Sham-operated rats served as sham group. JPYS (1.36 g/kg/d) was administered orally to CKD rats daily for six consecutive weeks. Results showed that JPYS treatment notably improved renal function and pathological injury in CKD rats. JPYS also restored the hematological parameters, including red blood cells, hemoglobin, and hematocrit. In parallel, the reduction level of EPO was reversed by JPYS. Furthermore, JPYS induced the accumulation of hypoxia inducible factor (HIF)-*α* protein expression. Collectively, these results provide convincing evidence for JPYS decoction in ameliorating CKD-associated anemia, and its mechanism might be related to regulate EPO production via HIF signaling pathway.

## 1. Introduction

Renal anemia is a common complication of chronic kidney disease (CKD) [1]. A relative deficiency of erythropoietin (EPO) production is the central cause that renal anemia develops [2]. Recombinant human EPO (rHuEPO) and erythropoiesis-stimulating agents (ESAs) are being applied to correct anemia in patients with CKD [3]. However, in the last decade, the ESA treatment-related harms, including increased mortality, cardiovascular events, and cancer progression, have raised our concerns and stimulated researchers' interest in finding alternative therapeutic approaches [4, 5]. Traditional Chinese medicine (TCM) has been widely used in China and other areas for centuries, which has been considered as an alternative medicinal purpose for a wide range of diseases, including the prevention and treatment of CKD and its associated complications, i.e., anemia [6–8]. Therefore, TCM is of great interest for being developed as a potential drug for treatment of CKD anemia.

Jian-Pi-Yi-Shen (JPYS), a Chinese herbal decoction, consists of Astragali Radix, Salviae Miltiorrhizae Radix et Rhizoma, Dioscoreae Rhizoma, Cistanches Herba, and other four ingredients. JPYS has been clinically prescribed to patients with CKD associated anemia for decades, as it is believed to possess the efficacies of fortifying the spleen, tonifying the kidney, activating blood, and resolving stasis. Previous pharmacological studies have supported that JPYS can improve renal function and kidney injury in CKD rats [9–11] and can stimulate the transcriptional expression of EPO in cultured kidney HEK293T cells [12]. Besides, the extract of Astragali Radix and Salviae Miltiorrhizae Radix et Rhizoma deriving from JPYS also has been found to ameliorate adenine-induced CKD rats [13]. These findings confirm the beneficial role of JPYS for treatment of CKD anemia. However, the molecular mechanism of JPYS in treating renal anemia still needs to be further studied.

The discovery of hypoxia-inducible factor (HIF) pathway in controlling EPO gene transcription has been regarded as a novel foundation that stimulates endogenous EPO production to promote physiologic erythropoietic response [14, 15]. Thus, HIF activation and increased production of endogenous EPO can be useful for therapeutic indications and manipulated for the treatment of renal anemia in CKD. Taking together, we speculate that JPYS could ameliorate renal anemia in CKD rats by targeting HIF-mediated EPO expression pathway. In this study, the improvement of anemia in CKD rats by JPYS treatment and the involvement of HIF signaling in JPYS-treated rats, including renal functions, hematological parameters, and EPO concentrations, as well as HIF activation, were investigated.

## 2. Materials and Methods

### 2.1. Preparation of JPYS Extract

JPYS extract was prepared as previously described [12]. In brief, eight herbs of JPYS were weighed and extracted in boiling water twice for 1 hour. After centrifugation, the supernatant was dried under reduced pressure to powder, and it was stored at -80°C. Before the treatment, the powder was redissolved with Milli-Q water and vortexed at room temperature.

### 2.2. Animals

Male Sprague-Dawley (SD) rats, eight weeks old, were purchased from Guangdong Medical Laboratory Animal Center (Foshan, China) and maintained in a specific pathogen-free (SPF) animal facility under a 12-hour light/12-hour dark cycle. Rodent food and drinking water were provided freely. All experiments were performed with protocols approved by the Institutional Animal Care Use Committee of Guangzhou University of Chinese Medicine and in accordance with National Institutes of Health Guideline for the care and use of laboratory animals (NIH Publications No. 80–23, revised 1996).

### 2.3. Induction of CKD Anemia Rats

The 5/6 nephrectomy was performed in two steps as previously described [9]. The sham group underwent the same operation consisting of laparotomy and manipulation of the renal pedicles, except for the destruction of renal tissue. All the surgical operation was performed under anesthesia with sodium pentobarbital (50 mg/kg body weight, intraperitoneal injection). The 5/6 Nx rats were randomly divided into two groups: rats without drug treatment (CKD group) and rats receiving JPYS treatments at dose of 1.36 g/kg/d (CKD + JPYS group). The JPYS extract was administered orally (by gavage). The same amount of distilled water was given to sham group (n = 6). After six weeks of treatment, all rats were euthanized. Blood samples were obtained from cardiac puncture. Kidneys were removed and preserved; one part of kidney was fixed in neutral formalin and embedded in paraffin for further histological analysis; another part was dissected in ice-cold PBS to remove the medulla and snap frozen in liquid nitrogen and stored at -80°C for further western blotting analysis.

### 2.4. Biochemical Analysis

Red blood cell (RBC), hemoglobin (Hb), and hematocrit (HCT) were executed using the Hematology Systems (Siemens 2021i, Erlangen, Germany), according to the manufacturer's instruction manual. Blood urea nitrogen (BUN), serum creatinine (Scr), and EPO levels were measured using ELISA kits according to the manufacturer's instructions (Thermo, Waltham, Massachusetts).

### 2.5. Histological Analysis

The extent of renal pathological injury was examined using periodic acid-Schiff (PAS) and Masson staining. The quantitative analysis approach was conducted as described previously [10, 16]. Briefly, tubular atrophy score in PAS staining was classified as follows: 0, normal tubules; 1, rare single atrophic tubules; 2, several clusters of atrophic tubules; 3, massive atrophy. Approximately 40-50 of glomerular tuft area in each rat and six rats per group were measured using Nikon NIS-Elements BR software (version 4.10.00, Nikon, Japan) as to quantify glomerular changes. The fibrotic area in Masson staining was determined by using Image J (NIH, Bethesda, MD). A minimum of 10 microscopic fields (200x) of each rat and six rats per group were captured. Atrophy score, glomerular change, and fibrotic area were measured randomly.

### 2.6. Western Blot Analysis

Proteins were extracted from snap-frozen kidney cortexes and were quantified with a Bio-Rad protein assay. Equal amounts of protein lysates were loaded and separated on 10% SDS-polyacrylamide gels and then transferred to nitrocellulose membranes or polyvinylidene difluoride membranes (Millipore, USA). Nonspecific proteins were blocked by incubating the membranes in 5% non-fat milk for 1 hour at room temperature. The membranes were then incubated with primary antibodies at 4°C overnight for specific proteins, followed by incubation with HRP-conjugated secondary antibodies for 1 hour at room temperature. HRP activity was visualized using Clarity Western ECL Substrate and a ChemiDoc MP Imaging System (Bio-Rad Laboratories, USA). Image Lab software version 5.1 was applied for densitometric analysis (Bio-Rad Laboratories, USA). The following primary antibodies were used in this study: polyclonal anti-HIF-2*α* from rabbit (Abcam; ab199; 1:200 dilution), polyclonal anti-HIF-3*α* from rabbit (Abcam; ab176464; 1:1000 dilution), and monoclonal anti-*β*-actin from mouse (Sigma; A5441; 1:5000 dilution).

### 2.7. Statistical Analysis

Data are expressed as Mean ± SEM. Statistical significance among groups was evaluated by one-way ANOVA and post hoc analysis with the Least Significant Difference (LSD) test or the Games-Howell test.* P* < 0.05 was considered statistically significant. All data were performed using SPSS statistics software (version 16.0, SPSS Inc., Chicago, IL, USA).

## 3. Results

### 3.1. JPYS Improves Renal Function of CKD Rats

Before the treatment of extract onto the animals, JPYS was chemically standardized [11]. The minimum requirement of identified chemical amounts for 1 g of dried extract powder of JPYS should be no less than the established parameters, i.e., sodium danshensu (0.45 mg/g); salvianolic acid B (1.80 mg/g); echinacoside (0.50 mg/g); calycosin 7-O-*β*-glucoside (0.68 mg/g); acteoside (0.10 mg/g); liquiritin (0.60 mg/g); astragaloside IV (0.05 mg/g); formononetin (0.60 mg/g). The extraction yield was ~32.59 ± 1.1% (w/w, Mean ± SD, n = 3). The JPYS extract being used in this study met the aforesaid requirements, which could guarantee the repeatability of biological results.

**Figure 1:**
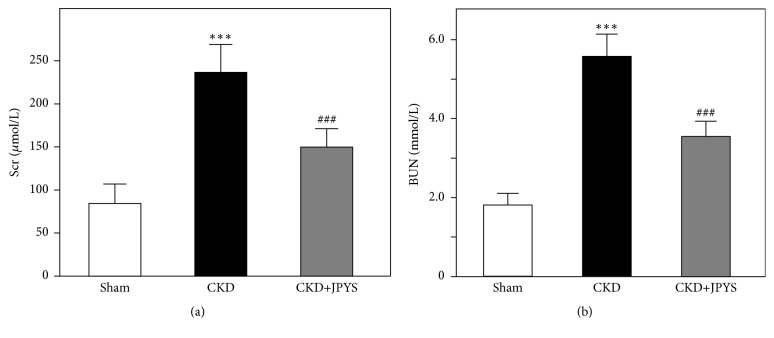
*JPYS improves renal function of CKD rats*. The levels of Scr (a) and BUN (b) were measured by ELISA. Values are expressed as the Mean ± SEM, where n = 6 rats per group (*∗∗∗ p* < 0.001 compared with the sham group; ^###^*p* < 0.001 compared with the CKD group).

To reveal the improvement of renal function by JPYS, the levels of BUN and Scr, two well-known indicators of renal function, were analyzed. Compared with sham group, rats in the CKD group showed significant higher BUN and Scr levels, indicating the decline of renal function. Compared with CKD group, treatment with JPYS extract robustly downregulated the BUN and Scr levels (Figure 1). In addition, the levels of plasma ALT and AST did not show significant difference among three groups (Supplementary Figure 1), indicating JPYS treatment would not cause hepatic toxicity in rats.

### 3.2. JPYS Attenuates Renal Pathological Injury in CKD Rats

**Figure 2:**
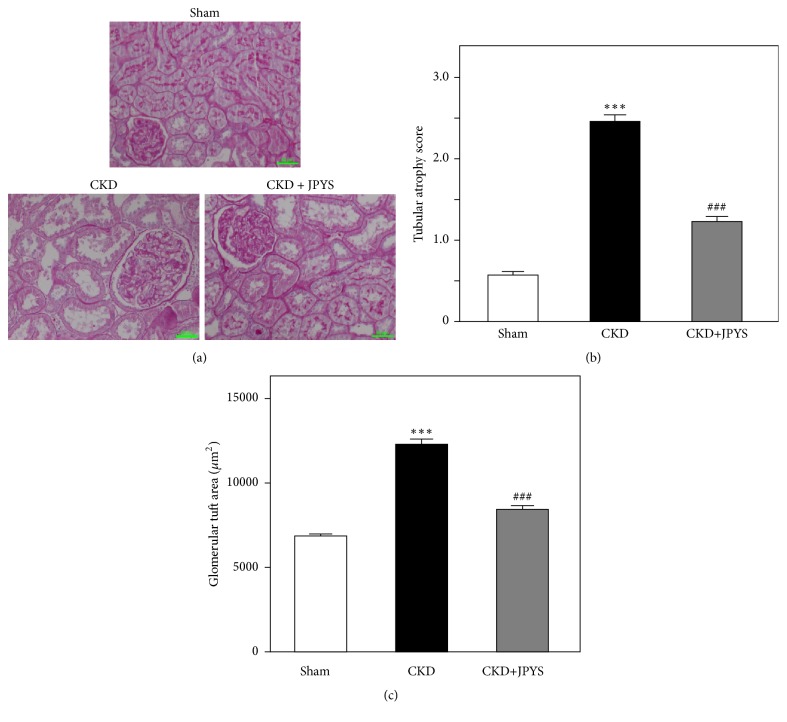
*JPYS attenuates renal tubular atrophy in CKD rats*. (a) PAS staining was employed to depict the kidney characteristics in each group. (b) Tubular atrophy score was quantified in each group. (c) Glomerular tuft area was quantified in each group. Representative images are shown at identical magnification, ×200, scale bar = 50 *μ*m. Data are presented as the Mean ± SEM, n = 6 rats per group (^*∗∗∗*^*p* < 0.001 compared with the sham group; ^###^*p* < 0.001 compared with the CKD group).

**Figure 3:**
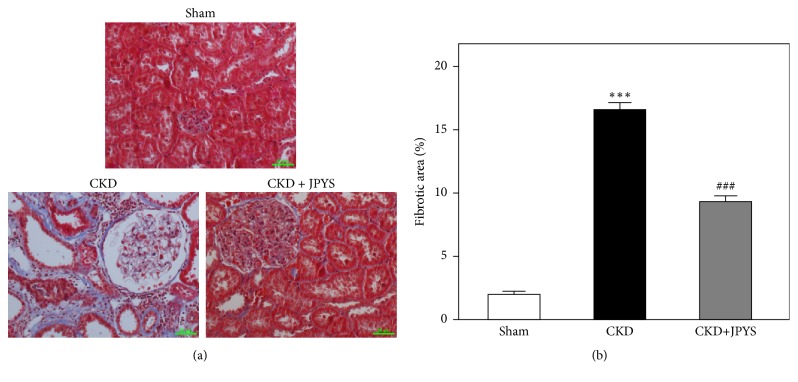
*JPYS reduces renal interstitial fibrosis in CKD rats*. (a) Masson staining was used to observe the renal tissue in each group. (b) Fibrotic area was quantified in each group. Representative images are shown at identical magnification, ×200, scale bar = 50 *μ*m. Data are presented as the Mean ± SEM, n = 6 rats per group (^*∗∗∗*^*p* < 0.001 compared with the sham group; ^###^*p* < 0.001 compared with the CKD group).

PAS staining showed that renal tubules appeared normal in the sham group with tubular atrophy score at ~0.5, yet the massive tubular atrophy was observed in CKD rats with increased tubular atrophy score at ~2.5. Compared with CKD group, the renal injury and tubular atrophy were alleviated in JPYS-treated group and that of score dropped to ~1.2 (Figures 2(a) and 2(b)). In addition, glomerular injury was also observed in CKD rats. Glomerular tuft area was robustly enlarged in CKD group compared with sham group, the area of which was sufficiently decreased after JPYS treatment (Figure 2(c)). Furthermore, Masson staining demonstrated that severe interstitial fibrosis from CKD group occurred, which was elevated more markedly than that of sham group. In JPYS treatment group of rats, the fibrotic area was significantly reduced as compared to that of CKD rats (Figures 3(a) and 3(b)).

### 3.3. JPYS Restores the Hematological Parameters of CKD-Induced Anemic Rats

**Figure 4:**
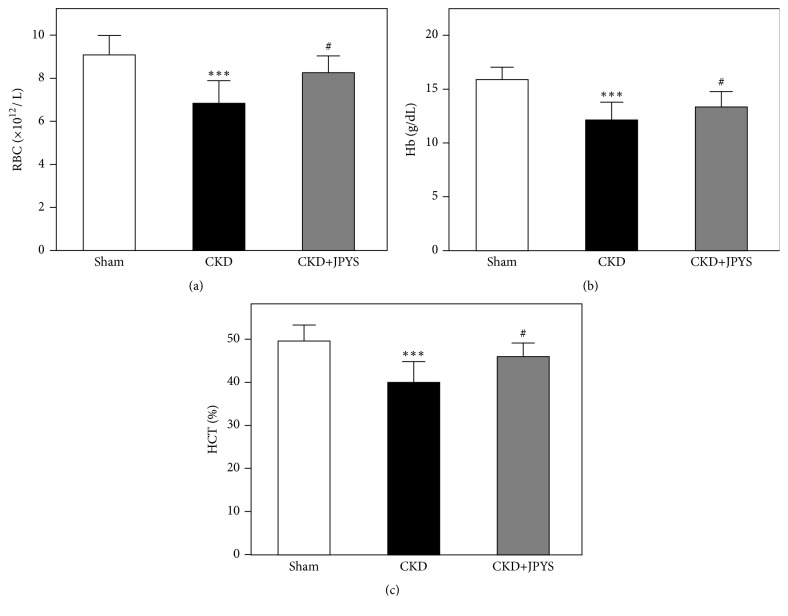
*JPYS restores the hematological parameters in CKD rats*. The levels of RBC (a), Hb (b), and HCT (c) were detected by ELISA kit. Values are expressed as the Mean ± SEM, where n = 6 rats per group (^*∗∗∗*^*p* < 0.001 compared with the sham group; ^#^*p* < 0.05 compared with the CKD group).

In Figure 4, the blood hematological parameters, including RBC, Hb, and HCT, were measured. Data showed that the levels of RBC, Hb, and HCT were statistically significant decreased in CKD-induced rats, confirming the 5/6 nephrectomy conduction successfully induced anemia in rats. For the CKD rats treated with JPYS extract, the decreased levels of RBC, Hb, and HCT were obviously restored, from 7.4 to 8.5×10^12^/L (*P*=0.03) in RBC, 12.4 to 13.9 g/dL (*P*=0.02) in Hb, and 38.9 to 45.6% (*P*=0.04) in HCT.

### 3.4. JPYS Induces the Production of EPO

**Figure 5:**
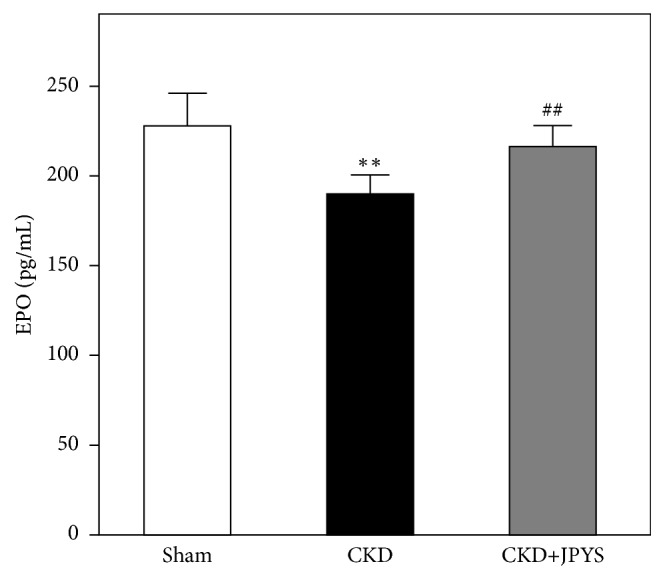
*JPYS stimulates EPO production in CKD rats*. EPO levels in each group were measured by ELISA. Values are expressed as the Mean ± SEM, where n = 6 rats per group (^*∗∗*^*p* < 0.01 compared with the sham group; ^##^*p* < 0.01 compared with the CKD group).

**Figure 6:**
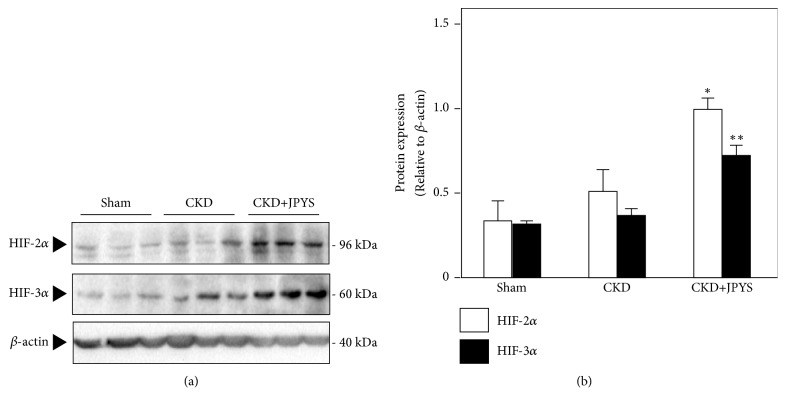
*Effects of JPYS on protein expressions of HIF-2α and HIF-3α in renal tissue of CKD rats*. (a) The protein lysates of kidney tissue were collected to determine the expressions of HIF-2*α* and HIF-3*α* by western blot. (b) Quantification of blot intensity for HIF-2*α* and HIF-3*α* was conducted. Representative western blot images are shown. Data are expressed as the Mean ± SEM, where n = 6 rats per group (^*∗*^*p* < 0.05 or ^*∗∗*^*p* < 0.01 compared with the CKD group).

Renal anemia is mainly considered to be less of EPO production from the kidney. To investigate the inductive effect of JPYS on EPO, the levels of endogenous serum EPO were determined by ELISA. EPO levels were dramatically dropped in CKD group, the levels of which could be significantly raised near to normal in JPYS-treated rats (Figure 5). We further performed western blot analysis to test the activation of HIF-*α* protein. In CKD group, the expressions of HIF-2*α* and HIF-3*α* were increased slightly as compared to sham group. The accumulations of HIF-2*α* and HIF-3*α* by JPYS administration were much stronger than that of CKD rats (Figure 6).

## 4. Discussion

Anemia, an almost irreducible complication of CKD, occurs more frequently in patients with advanced kidney dysfunction and relates to quality of life and mortality in CKD patients [17, 18]. Anemia in CKD is mainly due to inadequate amount of EPO production of injured kidneys, and the EPO deficiency is proposed to be the central feature of CKD associated anemia [1, 19]. HIF is a critical intermediate in the defense mechanisms against hypoxia and EPO is one of its target genes. Herein, targeting HIF stabilization in increasing more physiologic EPO levels offers a novel approach to improve the management of anemia [14].

In the present study, we investigated the effect of JPYS on improving renal anemia in CKD rats. JPYS significantly improves renal function and hematological parameters of CKD-induced anemic rats. These effects could be involved with the production of EPO via HIF signaling pathway. In line with these findings, our previous studies revealed that JPYS remarkably retards development and progression of CKD in animal model and induced expression of EPO in cultured kidney cells [9, 10, 12]. The results of this study demonstrated that the serum EPO levels were decreased in CKD rats. Consistent with our finding, Rahman et al. reported that the plasma EPO levels were remarkably reduced in animal models of renal anemia [20]. Yu et al. found that the mRNA expression of EPO was upregulated in rat remnant kidney [21]. We speculate that the variation of EPO levels in between plasma and kidney may be due to the progression stage of CKD. Indeed, the plasma EPO levels were changed during the development of CKD rats [20]. Similarly, acute renal injury increases EPO production at the beginning of the disease with a notable tendency to reduce just after the progress of injury [22].

HIF is a heterodimer comprising of an *α* and *β* subunit. The *α* and *β* subunits of HIF bind together in the cell nucleus to form a functional dimer, which results in activating transcriptional expression of erythropoietin [14]. In general, HIF-*β* is constitutively expressed and present excess, while HIF-*α* is subjected to ubiquitination and proteasomal degradation under normoxic conditions [19]. However, hypoxia causes HIF-*α* stabilization by inhibition of prolyl hydroxylase domain proteins. These findings represent a novel therapeutic application against anemia in chronic kidney disease [23]. HIF-*α* exists in three isoforms, HIF-1*α*, HIF-2*α*, and HIF-3*α*. In recent findings, HIF-1*α* plays a vital role in the response to local ischemia and has effects on angiogenesis and anaerobic metabolism [14]. HIF-2*α* is reported to be the key regulator of endogenous EPO gene transcription [24]. In agreement with this, our current study revealed that the protein level of HIF-2*α* and EPO was robustly upregulated by JPYS treatment, which supported JPYS would have benefits in anemia of CKD via HIF activation. However, the HIF-1*α* protein level was not detected in all experimental groups. We speculated that HIF-1*α* may play a role in the early period of CKD. In support of this, it was found that HIF-1 governed the response to hypoxia at the beginning and was degraded over time, whereas HIF-2 became main regulator and mediated chronic hypoxia [25, 26]. The roles of HIF-3*α* are less known, although it is generally considered as a negative regulator of HIF-1*α* and HIF-2*α* [27]. Recent findings suggest that there is a switch in HIF signaling, passing the signaling from HIF-1 to HIF-2 and finally to HIF-3 during prolonged hypoxia, where HIF-3 is considered to promote angiogenesis and long-term survival [25]. In present study, JPYS treatment induced the expression of HIF-3*α* protein level. Angiopoietin-1 is one of the important angiogenic factors for angiogenesis. Decreased angiopoietin-1 level has been identified and the angiopoietin-1/VEGF-A ratio was decreased in patients with CKD [28]. Future prospective studies are needed to examine whether HIF-3 can stimulate angiogenesis as to play a role in progression of CKD.

The anemia of CKD has been also reported to be associated with inflammatory cytokine levels [29]. The suppressive effects of proinflammatory cytokines on erythropoiesis are one of the key factors that cause the anemia of inflammation [30]. In our previous findings, JPYS regulated the cytokine expressions in cultured macrophages and 5/6 nephrectomized CKD rat [10, 12]. We speculate that the trophic role of JPYS in renal anemia may be also associated with inhibition of inflammation. In line with this, it has been found that the increased erythropoiesis via HIF signaling inhibits hepcidin expression, whereas the upregulated hepcidin levels in patients with CKD are associated with increased inflammatory activity [17, 31].

## 5. Conclusion

In conclusion, JPYS decoction could relieve renal anemia in 5/6 nephrectomized rats, which might be associated with upregulation of erythropoietin via hypoxia inducible factor signaling.

## Data Availability

The data used to support the findings of this study are available from the corresponding author upon request.

## Supplementary Materials

Supplementary Materials Supplementary Figure 1: Effect of JPYS on liver function in CKD rats. The levels of ALT (A) and AST (B) were measured by ELISA. Data are expressed as the Mean ± SEM, where n = 6 rats per group.
