# Supplementary material for: Jian-Pi-Yi-Shen Decoction Relieves Renal Anemia in 5/6 Nephrectomized Rats: Production of Erythropoietin via Hypoxia Inducible Factor Signaling
Source: Evid Based Complement Alternat Med. 2019 Mar 3;2019:2807926. doi: 10.1155/2019/2807926 (PMC6420977; doi:10.1155/2019/2807926)
Supplement: Supplementary Materials — Supplementary Figure 1: Effect of JPYS on liver function in CKD rats. The levels of ALT (A) and AST (B) were measured by ELISA. Data are expressed as the Mean ± SEM, where n = 6 rats per group. [file 2807926.f1.pdf]

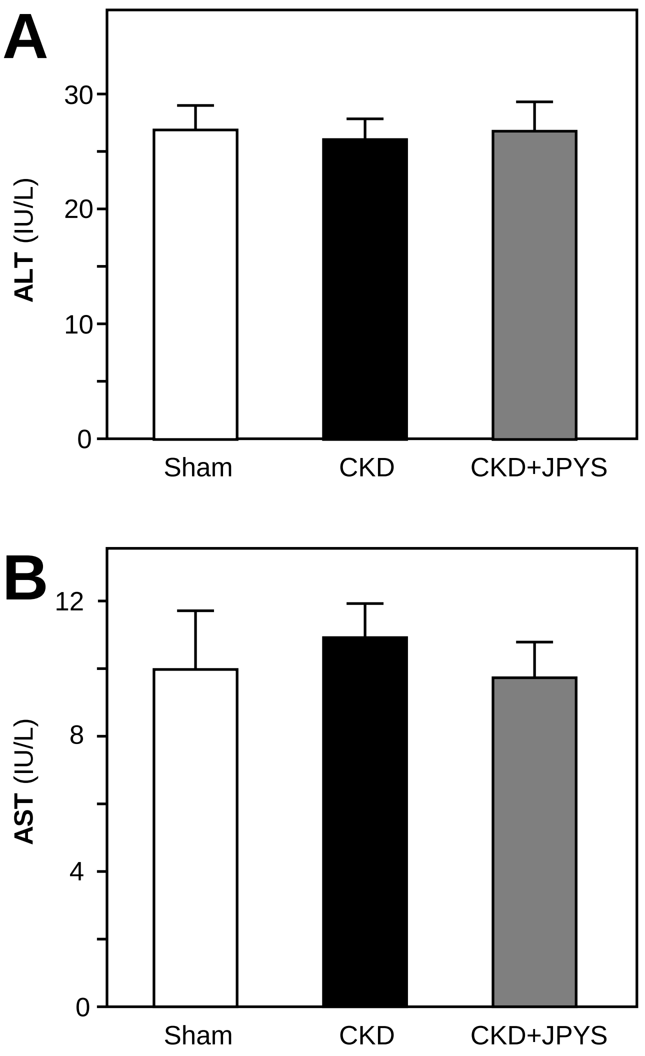

Supplementary Figure 1. Effect of JPYS on liver function in CKD rats.
The levels of ALT **(A)** and AST **(B)** were measured by ELISA. Data are expressed as the Mean ± SEM, where n = 6 rats per group.
